# 50 years of Iranian clinical, biomedical, and public health research: a bibliometric analysis of the Web of Science Core Collection (1965-2014)

**DOI:** 10.7189/jogh.08.020701

**Published:** 2018-12

**Authors:** Parisa Mansoori

**Affiliations:** Centre for Global Health Research, Usher Institute of Population Health Sciences and Informatics, University of Edinburgh, Edinburgh, Scotland, UK

## Abstract

**Background:**

A substantial growth has been reported in Iran’s number of clinical, biomedical, and public health research publications over the last 30 years. It is of interest to investigate whether this quantitative growth has also led to a larger number of papers with a high citation impact; to explore where the capacity for performing research lies; and which fields/institutions are lagging behind.

**Methods:**

This was a bibliometric study. Web of Science Core Collection and its different tools were used for retrieving and analysing the publications. Information about the journals was found on Journal Citation Reports^®^. Different types of collaborations across the highly-cited papers was investigated based on the affiliations, the characteristics of the language of the authors’ names, and the authors’ study and work backgrounds.

**Results:**

Iran’s number of clinical, biomedical, and public health research publications has substantially increased since 2000, a surge was seen in 2007, and the figure reached a peak in 2011. 11% of the publications were in Pharmacology Pharmacy; and the majority originated in Tehran University of Medical Sciences. Six of the 10 journals that had published the most were Iranian journals. H-index of publications had also increased over time (almost doubled between 2000 and 2010). 30.9% of the most-cited publications had only relied on Iranian resources (including 48 publications); had been published in journals with impact factors ranging between 0.4 and 8.3; and the majority were original basic sciences research.

**Conclusions:**

In Iran, a great capacity for research lies in clinical, biomedical, and public health fields which can be strengthened with further investment. It is important to use this capacity in a way that would align with the national population health needs. It is also essential to consider the limitations of only relying on bibliometric tools for assessing health research activities. Finally, the Iranian science policy-makers are encouraged to (i) support the researchers and institutions that have proved research capacity; (ii) direct further resources towards research areas and/or institutions that are lagging behind; (iii) facilitate further international collaboration with the academics and/or institutions that have shown the capacity for conducting successful research projects with Iran.

In recent decades, several low- and middle-income countries (LMICs) have become prominent contributors to the global scientific output [1]. For instance, although the largest proportion of public health research output continue to originate in North America and Western Europe, contribution of several countries from Eastern Mediterranean Region (EMR) and South-East Asia has greatly increased [2]. Iran is a remarkable example of such emerging scientific nations that had witnessed the fastest growth worldwide in publication counts, from 736 in 1996 to 13 238 in 2008 [3].

Since 1985, the governance of education and research in Iranian public universities of medical sciences (including 58 universities in 2017) [4] has been entrusted to the Ministry of Health and Medical Education (MOHME) [5]. Therefore, it is important to study Iran’s publications in clinical, biomedical, and public health research separately from other fields of science. Multiple studies have thus far reported the increase in Iran’s number of medical research publications in various areas, from general fields, eg, pediatrics [6] or dental research [7] to more specific areas, eg, breast cancer [8], diabetes [9], or the rationale use of drugs [10]. However, there is still a lack of an overall landscape of the changes across all fields of clinical, biomedical, and public health research. Furthermore, some criticize Iran’s research publications for having a very low citation impact and for having had increased only in the quantity [11,12].

A better understanding of Iran’s growth in research could first inform the national research policy-makers about that which researchers, institutions, and fields have achieved an acceptable capacity to perform and publish research, thus are worthy to be funded; and that which ones have lagged behind and could benefit from further capacity-building and/or investment. Second, the findings should help stakeholders from international organizations, eg, the World Health Organization, to better understand the local research capacity of Iran, which could be used in regional and/or global projects. Third, studying the collaborations that have led to highly-cited publications could assist both the Iranian and the international researchers and/or institutions to identify potential collaborators. Finally, a better insight into the changes in Iran’s health research output could also provide lessons to share with other LMICs who are to strengthen their health research capacity.

This study aims to use bibliometric methods to: (i) study the landscape of Iran’s clinical, biomedical, and public health publications over 50 years, and characterize some of the major trends; (ii): compute the changes of h-index of publications over this period to investigate whether the growth has been limited to the quantity; and (iii) identify the publications that contribute to Iran’s h-index, indicate which ones only relied on academics in Iran, and identify major characteristics of the publications.

## METHODS

The Methods section is structured in three parts, which each describes the methods that were used for addressing one objective.

### Methods for objective 1: Studying the landscape of publications

After considering the strengths and limitations of various international databases, eg, Google Scholar, Scopus, and Web of Science Core Collection (WoS CC) [13], WoS CC was chosen for the purpose of this study. It was chosen over Scopus for offering better tools to refine and analyse search results at the time the study was conducted. WoS CC, formerly known as Science Citation Index, is the oldest citation database, currently owned by Clarivate Analytics Company (Philadelphia, United States of America). WoS was accessed via the University of Edinburgh Library.

On February 28, 2018, a search was performed in WoS CC of all the publications from Iran between 1965 and 2014 by running an ‘Advanced Search’ with the country field tag: CU = Iran. To include papers with a topic relevant to clinical, biomedical, and/or public health, search results were refined by selecting 48 relevant items listed under the ‘Research Areas’ option on the refine panel (list of the included ‘research areas’ is provided in Table S1 in **Online Supplementary Document(Online Supplementary Document)**).

The goal was to study the longest possible period. The reason for which 1965 was chosen as the start year is that WoS currently only indexes the authors’ affiliations of publications since 1966, hence, articles published in earlier years cannot be captured by searching the country subfield [14]. Year 2014 was chosen for end of the period because once a new journal is indexed in WoS CC, the most recent three years of the journal’s back issues will be obtained by WoS [15], which increases the figures. Thus, it is better to exclude the publications of the last three years in such bibliometric analyses. The impact factor (IF) and country of origin of the journals were found on Journal Citation Reports^®^ (JCR). The approach to analysing the publications is detailed in **Box 1****.**

Box 1 **Approach to analyse the retrieved research publications**

Retrieved records were analysed using the “Results Analysis” feature of WoS CC and were ranked in a descending order in the following fields: ‘Countries/Territories’; ‘Organization-Enhanced’; ‘Source Titles’; ‘Research Areas’; ‘Authors’; and ‘Document Types’. While most of these fields are self-explanatory, it is worth mentioning that ‘Organization-Enhanced’ that indicates authors’ affiliations, comprises the unified and most accurate name variant of addresses [16]. It should also be noted that regarding prolific authors, it is possible that some publications that belong to authors with the same surname and initial are incorrectly attributed to one author. Therefore, an ‘Advanced Author Search’ was performed to ensure the publications linked to prolific authors belonged to one person. To attain the landscape of the growth across different research areas, the number of publications in each year and in each research area was counted by first, retrieving the publications in every year and second, analysing them for research areas. Finally, the data were exported to an Excel file for calculations.

### Methods for objective 2: Investigating the changes in h-index of publications

H-index is a bibliometric indicator developed in 2005 and originally intended as a measure of the quantity and the citation impact of an individual’s research output, trying to capture both in a single number [17]. However, it can be adapted to assess the characteristics of research from both institutions and countries and provide an understanding of their capacity for research [18]; in such indications, it can be called h-core [19]. This analysis only included citations from sources that were indexed in WoS CC. The approach to identify the 5-year h-indices is explained in **Box 2****.**

Box 2 **Approach to identify the 5-year h-indices**

To assess the changes in h-index of publications over time, the method that Badenhorst et al had introduced for assessing the global public health research capacity was followed [2]. H-indices were calculated over ten 5-year periods: 1965-1969; 1970-1974; 1975-1979; 1980-1984; 1985-1989; 1990-1994; 1995-1999; 2000-2004; 2005-2009; and 2010-2014. To minimize the expected lag between publications and getting cited [20], a ‘citation window’ of three years following each 5-year period was added [2]. This means that, for instance, when calculating the h-index for the 5-year period between 2000 and 2004, publications with dates 2000-2004 were included, while all the citations that those publications had received in the period 2000-2007 were taken into account in calculating the h-index. The search criteria were the same as the ones described for objective 1, except for the time span. Once the search was complete for each 5-year period, citation information of the articles were found using the ‘Citation Reports’ feature of WoS CC. “Citation Reports” shows the number of citations that every search result has received every year. To include the 8-year citations, the time span of the search was set for the 8-year period, while after running the search and before creating the “report”, the publication years were refined to only include the documents that were published within our desired 5-year period. To calculate the h-index, all the citation data was exported to a Microsoft Excel (Microsoft Inc, Seattle WA, USA) file, then the total citation counts of each document in an 8-year period was calculated, and the documents in each 5-year period were ranked by citation counts in a descending order and documents were numbered accordingly. H-index was computed by finding where the rank was lower or equal to its corresponding citation count.

### Methods for objective 3: Identifying the h-core publications and investigating their characteristics

**Figure 1:**
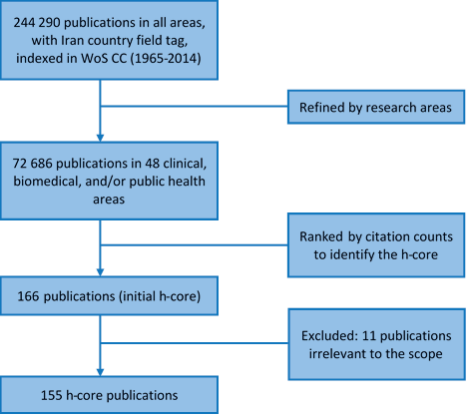
Search strategy to identify the h-core publications.

The same search strategy used for addressing Objective 1 was applied and the retrieved publications were ranked by citation counts in a descending order for identification of h-index (**Figure 1**). Citations were counted until the time of conducting the search (February 28, 2018). By using the ‘Marked List’ feature of WoS, the h-core papers were selected for further analysis. In WoS, the allocation of articles to research areas is done automatically based on the scope of the journals where the articles are published. Hence, through refining the search results by ‘Research Areas’ some articles may appear that were published in clinical, biomedical, and/or public health journals, while their content may not be fully relevant. To ensure that the research area of the included h-core papers was in clinical, biomedical, and/or public health, the titles, abstracts, and when necessary full-texts of the records were screened and the ones with irrelevant topics were excluded. Excluded papers were replaced with their following publications, and this was done until the rank was lower than or equal to the citation counts (ie, h-index). Initially, there were 166 records which finally reached 155 records after excluding the irrelevant papers: h-core = 155. Different types of collaborations in the h-core papers were investigated as explained in **Box 3**.

Box 3 **Approach to find where the authors came from**

Different types of collaborations were investigated by looking at the affiliation of the authors and their country of origin (to identify whether it was Iran or not). Authors’ affiliations were found in the full-text of papers, while to explore the country of origin of authors, language of their names and also study/work background of the authors were investigated by searching the Web. Most Persian names have distinguishable characteristics. The author, who is a native Persian speaker, investigated the language of the names and categorized the articles as explained below:

-Authors with Persian names who only had Iranian affiliation(s) were considered as “Iranian in Iran”.
-Authors with Persian names who only had non-Iranian affiliation(s) were considered as ‘Iranian abroad’. Since there might be people with Persian first and surnames who had never studied nor worked in Iran, education and work background of authors with Persian names but international affiliation(s) were also searched on the Web.
-Publications of authors with Persian names but with dual affiliations (Iranian and international affiliations) were considered as a collaboration of “Iranian in Iran and Iranian abroad”.
-Authors with non-Persian names and non-Iranian affiliation(s) were considered as ‘International’.
-Authors with non-Persian names, but with Iranian affiliation were considered as ‘Foreigner in Iran’.
-Articles which were clearly part of a large international collaborative project, funded by international organizations, and/or with collaborators from various parts of the world, and/or on topics that international collaboration was inevitable were considered as “Consortium”.

The 155 h-core papers were analysed for: (i) authors; (ii) organization-enhanced; (iii) journals; (iv) document types; (v) research areas; and (vi) collaborating countries. A subset of the h-core papers comprising ‘only Iranian’ publications, meaning papers which solely had Iranian authors affiliated with Iranian institutions, was analysed for the first five abovementioned fields. These papers were also categorized into basic, clinical, and public health research according to their content. The journals where the ‘only Iranian’ papers had been published were further analysed using JCR. To identify what proportion of the citations to each of the ‘only Iranian’ h-core papers originated in Iran, first, a ‘Cited Reference Search’ was performed to find items that had cited each of the papers. Second, by running an ‘Advanced Search’ with the country field tag, all the publications with at least one author based in Iran were retrieved. A combination of these two searches provided the citing documents that had at least one author in Iran, and the proportion of these publications to all citations was computed.

## RESULTS

The Results section is structured in three parts, which each describes the results for one objective.

### Results for objective 1: A landscape of clinical, biomedical, and public health publications (1965-2014)

**Figure 2:**
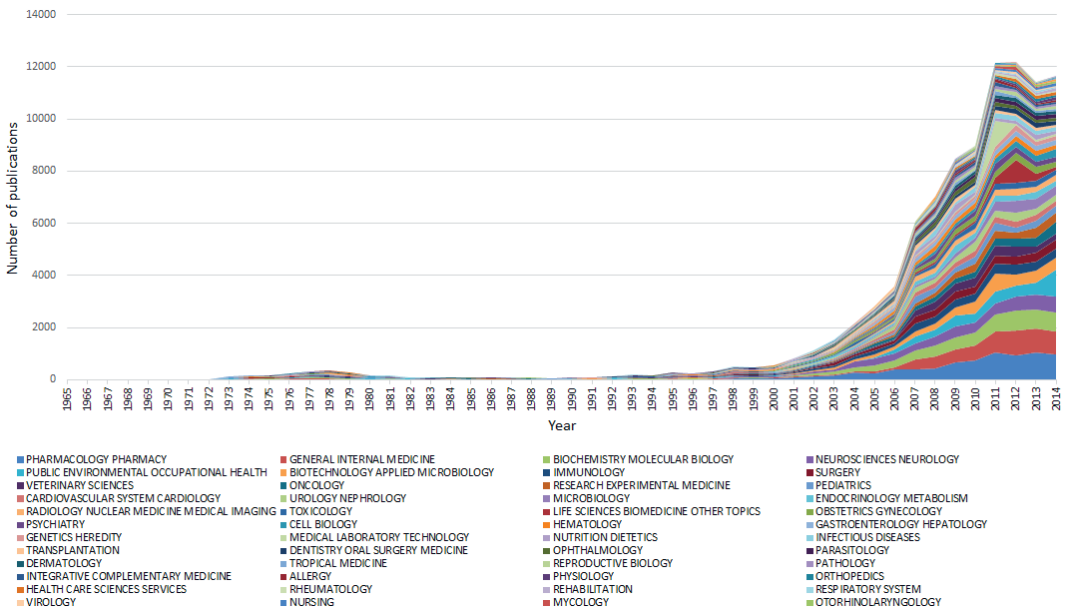
Iran's publications across clinical, biomedical, and public health research areas (1965-2014, indexed in Web of Science Core Collection).

On February 28, 2018, there were 244 290 research publications indexed in WoS CC with at least one author with an Iranian affiliation for the period 1965-2014; a total of 72 686 (29.7%) were in clinical, biomedical, and/or public health. The absolute number of publications in each year, and the annual and the total number of publications in each research area are provided in **Online Supplementary Document(Online Supplementary Document)**. **Figure 2** illustrates the landscape over 50 years.

A substantial increase is evident in the quantity of publications over 50 years, rising from only 1 publication per year in the late 1960s to a total of 8984 in 2014. An overall increase has started since 2000, and has become more substantial between 2006 and 2007 (increasing from 3587 to 6058), and another surge has occurred between 2010 and 2011. The growth peaked in 2011, reaching 9646 publications, remained relatively steady in 2012, while decreased to 8616 in 2013. It should be noted that the number of publications that are illustrated in **Figure 2** are the summed up number of publications in different research areas, meaning that if a record had been assigned to multiple research areas by WoS, it had been counted more than once. That is why the number of publications indicated in **Figure 2** are higher than the annual number of publications.

Results of analyses of the publications and information about the journals where the highest proportions of papers have been published are summarized in **Table 1**. IF of the 10 journals where the majority of papers were published ranged between 0.57 and 2.4; six of them were Iranian journals; and one of these 10 journals is no longer indexed in WoS CC (ie, Life Science Journal - Acta Zhengzhou University Overseas Edition). Ten percent of the international collaborations has been with the USA, the UK, and Canada. The majority of publications were original articles (69.0%), followed by meeting abstracts (20.4%), letters (4.0%), and review articles (2.7%). The full analysis results is available in **Online Supplementary Document(Online Supplementary Document)**.

**Table 1:** Results of analysis of 72 686 Iranian clinical, biomedical, and public health publications (1965-2014), indexed in Web of Science Core Collection, representing: research areas with most publications, the most prolific organizations and authors, journals that had published the majority of publications, and countries with which Iran has had the most collaboration*

Rank	Research areas with most publications	Prolific organizations†	Prolific authors‡	Journals that had published the most	Countries with most collaborations§
**1**	Pharmacology Pharmacy (8167)	Tehran University of Medical sciences (17 401)	Abdollahi, M (558)	Iranian Journal of Public Health (1442) – From Iran –IF in 2016: 0.76	USA (3684)
**2**	General Internal Medicine (6075)	Shahid Beheshti University of Medical sciences (6728)	Azizi, F (491)	Iranian Red Crescent Medical Journal (1146) – From Iran – IF in 2016: 0.86	UK (2331)
**3**	Biochemistry Molecular Biology (5844)	University of Tehran (6086)	Larijani, B (447)	Journal of Research in Medical Sciences (1005) – From Iran – IF in 2016: 1.2	Canada (1317)
**4**	Neuroscience Neurology (4638)	Shiraz University of Medical Sciences (5241)	Rezaei, N (429)	Life Science Journal - Acta Zhengzhou University Overseas Edition (1001) – From China – Indexed between 2008-2012	Germany (1050)
**5**	Public Environmental Occupational Health (4353)	Isfahan University of Medical Sciences (3876)	Dehpour, AR (428)	Clinical Biochemistry (999) – From Canada – IF in 2016: 2.4	Australia (1020)
**6**	Biotechnology Applied Microbiology (3712)	Pasteur Institute of Iran & Le Réseau International des Instituts Pasteur (RIIP) combined (3807)	Zarrindast, MR (378)	Archives of Iranian Medicine (931) – From Iran – IF in 2016: 1.2	Sweden (736)
**7**	Immunology (3321)	Tarbiat Modares University (3732)	Malekzadeh, R (353)	African Journal of Biotechnology (694) – From Kenya – IF in 2016: 0.57	France (693)
**8**	Surgery (3256)	Mashhad University of Medical Sciences (3588)	Alavian, SM (345)	European Psychiatry (642) –From France – IF in 2016: 3.1	The Netherlands (675)
**9**	Chemistry (3182)	Tabriz University of Medical Sciences (3331)	Zali, MR (344)	Iranian Journal of Pharmaceutical Research (619) – From Iran – IF in 2016: 1.5	Italy (666)
**10**	Veterinary Sciences (3017)	Iran University of Medical Sciences (2372)	Ghavamzadeh, A (340)	Iranian Journal of Pediatrics (597) – From Iran – IF in 2016: 0.7	Malaysia (626)

IF – impact factor

*The numbers in brackets show the number of the documents, based on the data from 72 686 publications in clinical, biomedical, and public health research areas (1965-2014) with at least one Iranian affiliation

†7474 documents were attributed to the Islamic Azad University, a very large private university with many branches all across Iran, which all seem to be using the same affiliation. It was impossible to distinguish the publications that originate in each branch, therefore, the Islamic Azad University was excluded from the results. Furthermore, in addition to Pasteur Institute of Iran, Le Réseau International des Instituts Pasteur (RIIP) was among the most productive organizations. Since the publications attributed to both of these two institutions belonged to one organization, the number of their publications were summed up.

‡The names of two authors from the top list were excluded: Amini, M; and Karimi, M, which are both a very common combination of a surname and an initial among Iranians. The reason for exclusion was that an ‘Advanced Author Search’ revealed the records that were attributed to these two authors included publications of multiple authors with the same surname and initial.

§Web of Science reports publications affiliated with England, Scotland, Northern Ireland, and Wales separately. Here, the summed up figure is reported for the UK.

### Results for objective 2: The changes in h-index of publications (1965-2014)

**Table 2** summarizes the changes in h-index. In general, the 5-year h-index has had a growing trend over the last 5 decades, and it has become ~ 1.5 times greater every 5 years between 1990 and 2004. The 5-year h-index of publications between 2005 and 2009 was more than doubled compared to the figure for publications between 2000 and 2004 (rising from 35 to 78). The h-index of documents published during 2010-2014 reached 105, meaning that within this period 105 documents were published that each one had been cited at least 105 times until the date of conduct of this study.

**Table 2:** Five-year h-indices of Iranian clinical, biomedical, and public health publications (1965-2014), indexed in Web of Science Core Collection

Number	5-year period	5-year h-index
**1**	1965-1969	0
**2**	1970-1974	9
**3**	1975-1979	16
**4**	1980-1984	13
**5**	1985-1989	10
**6**	1990-1994	14
**7**	1995-1999	21
**8**	2000-2004	35
**9**	2005-2009	78
**10**	2010-2014	105

### Results for objective 3: Identifying the h-core publications and identifying their characteristics

Bibliographic information of the h-core publications with their citation counts are provided in Table S2 in **Online Supplementary Document(Online Supplementary Document).** Citation counts ranged between 156 and 3959. The most-cited paper was a systematic analysis for the Global Burden of Disease (GBD) Study 2010, with contribution from 118 institutions (two of which were Iranian) and was published in 2012 in *The Lancet*. The oldest highly-cited record (published in 1973) was authored by three Iranians affiliated with Namazi Hospital of Pahlavi University, and one non-Iranian person with the same Iranian affiliation. **Table 3** summarizes the major results from analysis of the 155 h-core papers and the full results are provided in **Online Supplementary Document(Online Supplementary Document)**. Twelve international institutions appeared among the top contributors to the h-core papers, while the three Iranian ones included Tehran University of Medical Sciences – TUMS (51 papers), Shahid Beheshti University of Medical Sciences (19 papers), and Isfahan University of Medical Sciences (13 papers).

**Table 3:** The top contributors to the 155 Iranian clinical, biomedical, and public health h-core publications (1965-2014), indexed in Web of Science Core Collection*

Rank	Research Areas with ≥6 h-core papers	Organizations† contributed to ≥13 h-core papers	Authors (from Iran, contributed to ≥5 h-core papers)	Journals that have published ≥3 h-core papers	Collaborating Countries
**1**	General Internal Medicine (31)	Tehran University of Medical Sciences (51)	Farzadfar, F (9)	The Lancet (17)	UK (74)
**2**	Pharmacology Pharmacy (18)	University of London (29)	Naghavi, M‡ (8)	Journal of Endodontics (7)	USA (65)
**3**	Oncology (13)	Harvard University (24)	Forouzanfar, MH‡; Torabinejad, M‡ (7)	New England Journal of Medicine (5)	Canada (35)
**4**	Biochemistry Molecular Biology (12)	University of California System (22)	Abdollahi, M; Azizi, F; Ezzati, M‡; Kelishadi, R; Pourmalek, F‡ (6)	Advanced Drug Delivery Reviews; American Journal of Clinical Nutrition; Biosensors Bioelectronics; Diabetes Care; Journal of Allergy and Clinical Immunology; Nature Genetics (3)	People’s Republic of China; Sweden (27)
**5**	Biotechnology Applied Microbiology; Endocrinology Metabolism; Nutrition Dietetics (9)	University College London (20)	Azadbakht, L; Esmailzadeh, A; Malekzadeh, R; Mehrabi, Y (5)		Australia; Brazil; France; Germany; India (24)
**6**	Public Environmental Occupational Health (8)	Shahid Beheshti University of Medical Sciences (19)			The Netherlands (22)
**7**	Cell Biology; Chemistry; Dentistry Oral Surgery Medicine; Genetics Heredity (7)	Karolinska Institutet (18); National Institute of Health (NIH USA) (18)			Italy; Japan; South Africa; Switzerland (21)
**8**	Immunology; Neuroscience Neurology; Psychiatry; Science Technology other Topics (6)	Imperial College London; University of Washington (17)			Spain (20)
**9**		University of Oxford; University of Toronto; (15)			Argentina (18)
**10**		Isfahan University of Medical Sciences; Monash University; University of Sydney (13)			Colombia; Turkey (16)

*The names grouped in one cell have had equal number of contributions, as presented in brackets.

†The same numbers were double reported for ‘University of Washington’ and ‘University of Washington Seattle’. Here, the figures are reported for ‘University of Washington’ as its preferred name variant.

‡Authors who at least in one of their publications were affiliated with international institutions.

**Figure 3:**
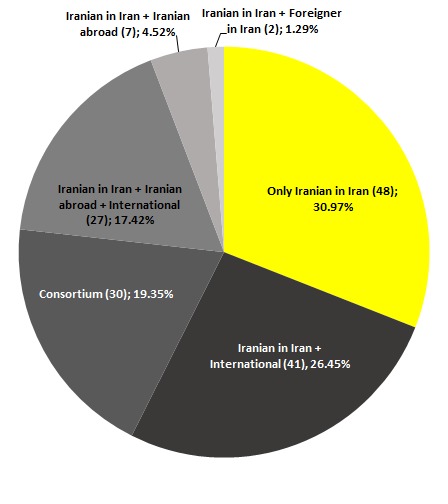
Distribution of different types of collaborations across the 155 Iranian clinical, biomedical, and public health h-core publications (1965-2014), indexed in Web of Science Core Collection.

Using the approach described in **Box 1**, different types of collaborations were identified as indicated in **Figure 3**. Of the total 155 records, 48 (30.9%) papers were ‘only Iranian’ and the rest, included some sort of international collaboration. Table S3 in **Online Supplementary Document(Online Supplementary Document)** summarizes the titles and bibliographic information of the 48 ‘only Iranian’ publications, the citation counts, and the proportion of citations to each that originated in Iran. The research areas, institutions, and authors with most contribution to the 48 “only Iranian” papers and the journals which have published at least two of these papers are listed in **Table 4**.

**Table 4:** The top contributors to the 48 “only Iranian” clinical, biomedical, and public health h-core publications (1965-2014), indexed in Web of Science Core Collection

Rank	Research areas with ≥3 papers	Organizations contributing to ≥3 papers	Authors contributing to ≥3 papers	Journals that have published ≥2 papers
**1**	Pharmacology Pharmacy (14)	Tehran University of Medical sciences (17)	Azizi, F (5)	Biosensors Bioelectronics (3)
**2**	Biochemistry Molecular Biology (7)	Shahid Beheshti University of Medical sciences (9)	Abdollahi, M; Mirmiran, P (4)	Diabetes Care; Journal of Theoretical Biology; Phytotherapy Research (2)
**3**	Biotechnology Applied Microbiology; Chemistry (6)	Tarbiat Modares University (7)	Kelishadi, R; Mohabatkar, H (3)	
**4**	Neuroscience neurology (4638)	Shiraz University (5)		
**5**	Science Technology other Topics (5)	Mazandaran University of Medical Sciences (4)		
**6**	Endocrinology Metabolism; Research Experimental Medicine (4)	Isfahan University of Medical Sciences; Ministry of Health and Medical Education; Shiraz University of Medical Sciences (3)		
**7**	Biophysics; Electrochemistry; Oncology; Psychiatry; Toxicology (3)			

The most-cited ‘only Iranian’ paper was a review article in basic sciences entitled “Hydrogel nanoparticles in drug delivery”, published in 2008 in *Advanced Drug Delivery Reviews*. The paper was a collaboration between three authors affiliated with Shiraz and Zanjan universities of medical sciences. It was cited 778 times, and only 9.1% of its total citations were from sources affiliated to Iranian institutions. In general, the proportion of citations that originated in Iranian institutions ranged between 0.8% and 97.7%. All of the six papers which over 80% of their citations was Iranian were in public health. While five of them were national and sub-national epidemiologic studies (addressing cancer; mental health; non-communicable diseases; cardiovascular risk factors; and metabolic syndrome), one was the preliminary results of a community-based programme for the prevention and control of cardiovascular diseases. The papers which had less than 10% of their citations originated in Iran (including 17 papers) were predominantly in basic sciences (16 out of 17).

The majority of the ‘only Iranian’ h-core papers were basic research (64.5%). Also, 62.5% of the 48 papers were original articles; and the remaining were review articles (further details in Supplementary Table S4 in **Online Supplementary Document(Online Supplementary Document)**). The 48 ‘only Iranian’ papers were published across 43 journals. IF of these journals ranged between 0.4 and 8.3 with a median of 2.3 (based on the IF in the year when each paper was published). A full list of the 43 journals where the ‘only Iranian’ papers had been published and their IFs are available in **Online Supplementary Document(Online Supplementary Document)**.

## DISCUSSION

This study was successful in providing a 50-year overview of Iran’s clinical, biomedical, and public health research publications that are indexed in WoS CC. It also identified the year in which the growth peaked; and characterized main trends across the publications. Furthermore, this paper suggested that the rise in the quantity to some extent has led to improvements in citation impact. Finally, it identified a set of the most-cited Iranian clinical, biomedical, and/or public health publications and highlighted where the capacity for producing highly-cited papers lies.

Several bibliometric studies had previously reported the substantial quantitative growth of Iranian research publications over the last three decades [3,21-23]. This paper visualized the changes in the number of Iranian clinical, biomedical, and public health research publications over 5 decades and found a significant rise until 2011 and a drop afterwards. Some of the proposed possible contributory factors to the quantitative increase in Iran’s research publications are the following: improved economy after recovery from the Iran-Iraq war (1980-1988) [11,24]; increased number of universities, research centers, students and faculty members; multiplication of postgraduate programs [11,21,25]; requiring students to publish papers to get their degree; providing academics with financial rewards per publication [11,26]; increased number of Iranians who study abroad and maintain international collaborations after returning to Iran [24]; or improved access to data sources [27].

Even if such assumptions justify the overall research growth in Iran, still the accelerated increases in certain periods require further explanation. For example, one surge is evident in 2007, which could partially be explained by the addition of a significant number of regional journals to the WoS CC, between 2005 and 2010 [28]. Regional journals were defined as journals publishing outside the US or the UK and containing the scholarship of authors from a particular region or country, and covering topics of regional interest [28]. Within that 5-year period, Thomson Reuters – the former owner of the WoS – indexed 1600 new regional journals that met the standard editorial criteria of the WoS [28], while concurrently, MOHME was supporting Iranian medical journals to improve their quality to meet international editorial and publishing standards [29]. Consequently, between 2005 and 2010, the number of Iranian journals indexed in WoS CC increased from only five to 41; 15 of the new additions were in clinical medicine [28]. Furthermore, once a new journal is indexed in WoS CC, the most recent three years of the journal’s back issues would also get indexed [15], and this suddenly adds up to the number of indexed documents. Finally, looking at the top-10 journals where the majority of Iranian clinical, biomedical, and public health papers have got published, six of the journals were Iranian. Parallel increases in the number of research publications and the number of journals that are indexed in international databases had been reported from other countries, eg, Brazil [30]. In sum, the addition of Iranian journals to WoS CC seems to have had contributed to the 2007 surge.

Another substantial rise in research output occurred between 2010 and 2011. Considering an often three- to four-year time lag between the initiation of research projects and their publication, the contributory factors to the 2011 peak should be traced back in a few years earlier. As such, the surge could, for instance, be associated with that Iran had its highest gross domestic expenditure on R&D (GERD) – ie, 0.75% – in 2007 [31]. This may have led to increased resources allocated to health research. Another possible contributor to this surge could be that, in 2009, a policy document entitled “Iran’s Comprehensive Scientific Map” was developed and released by ‘Iran’s Supreme Council of Cultural Revolution’ [32,33]. This council holds the highest level of authority for setting education and research policies in Iran. The so-called ‘Scientific Map’ provided a set of goals, policies, and requirements for development of science, technology and innovation system in Iran [33]. It partially outlined the targets by meeting which Iran presumably must achieve its broader ambition: ie, becoming the Middle East’s leading country by 2025 based on scientific and technological indicators [4,32]. Examples of targets included increased number of research centers, faculty members, and PhD students [31]. Investigating the impacts that implementation of this ‘Map’ may have had on clinical, biomedical, and public health research in Iran is of much interest.

Some correspond the drop in publication counts in 2013 to the tightening up of the economic and banking sanctions imposed on Iran [34]. Regarding the peak that was seen in 2011, it should be noted that this study included the publications by the end of 2014 and it is likely that publication counts have already exceeded the figures for 2011, or may exceed it in the future. Analyses of the data retrieved through PubMed show a continued growing trend until 2015 [31].

In terms of the most prolific institutions, TUMS stands on top with nearly three times as many publications as the next institution, ie, Shahid Beheshti University of Medical Sciences. As bibliometric analyses’ results are often not adjusted by the size of the unit (eg, by the number of students or academics), it is anticipated that TUMS – ie, the largest Iranian university of medical sciences – would lead the national ranking tables for output [35]. More importantly, TUMS, has an excellent reputation for medical education and research among most Iranians. Many top students and competent early-career academics intensively compete for admission in TUMS [36], therefore it is possible that the researchers at TUMS are potentially more productive than their peers. It could also be hypothesized that TUMS – which is based in the capital city Tehran and in many periods has been directed by leading scientists with strong networks inside and outside academia – may have been closer to the sources of funding. It is worth mentioning that the top-3 prolific Iranian universities of medical sciences also have the highest number of research centers, which could be another contributory factor to greater research publication productivity [4].

Having known the work background of the prolific authors, it seems that the majority of the top-10 are either (i) well-reputed mentors who attract many student projects that could lead to publications; and/or (ii) had held executive roles that has either facilitated access to resources, and/or has provided them with the necessary skills to initiate and effectively manage teamwork. Among the top-5 prolific authors two are pharmacologists, another two are clinicians in endocrine and metabolic disorders, and one is a pediatric immunologist. The landscape for research areas with most publications has been fairly similar. Pharmacology was the research area with the largest proportion of publications (11%), followed by General Internal Medicine, and Biochemistry Molecular Biology, each comprising ~ 8% of the total publications.

It could be suggested that Pharmacology and Biochemistry Molecular Biology are fields that most of their publications originate in schools of pharmacy. Schools of pharmacy in Iran seem to be more research-oriented than schools of medicine. This assumption is partially made based upon the structure of MOHME, where the schools of medicine are not only responsible for medical education and research, but also for providing health care services [37]. It has been suggested that the workload of medical faculty members and students within this structure may not leave sufficient time for research [37].

In terms of document types, it is interesting that following original articles (comprising 69.0% of total publications), the second most common document type was ‘meeting abstract’ (20.4%). This could be a result of the support and incentives that MOHME has been providing to promote participation of Iranian academics in international conferences [38]. In the research performance assessment of the universities of medical sciences that MOHME runs annually, accepted abstracts in international conferences provide points [39].

It is noticeable that the journals where the larger proportions of Iranian research had been published generally had relatively low IFs (0.57-2.4). It could be hypothesized that Iranians have found easier ways for publishing papers, eg, by submitting to Iranian and/or international journals with possibly higher acceptance rates. Other countries, eg, Turkey and Australia, had previously reported a decrease in the average IF of the journals where the total national publications were getting published after implementation of policies that strongly emphasized the value of the quantity of publications [40,41]. On the other hand, it could be argued that increased visibility of publications in Iran, even if achieved by publishing in mediocre journals, could have benefits. First, partaking in the international scientific community by publishing could provide junior researchers with a sense of accomplishment and encourage the competent ones to improve their research activities. Second, it assists international institutions and researchers to identify potential Iranian collaborators, which could lead to opportunities for exchange of knowledge, expertise, and resources [35]. Last but not least, visibility of publications to a larger group of peers attracts wider criticism which could lead to early detection of possible problems. For instance, the international criticism that was directed to the Iranian cases of alleged research misconduct, urged the national science policy-makers to take further steps for addressing issues regarding research and publishing integrity in Iran’s academia [12,42]. Such feedback would also promote discourse on research ethics in Iran, which in the long term could strengthen its growing scientific community [42].

The majority of international collaborations has been with the USA, the UK, and Canada, which – interestingly – all the three have had fairly challenging international relations with Iran over the last four decades. While the negative impacts that the imposed trade sanctions have had on Iran’s research activities should not be overlooked [34,43-45], it seems that international scientific collaborations had been established and/or maintained regardless of the political atmosphere [46]. Also, these countries are three of the most common destinations that Iranians choose for studying and/or immigration [47]. The ones who return to Iran after completion of their academic programs may continue collaboration with their former supervisors, or with the international network which they have established [24], while those who emigrate may continue collaborating with colleagues back home. Another point is that many of research consortiums are led by American and/or British institutions, and publications arising from such projects, if having Iranian collaborators too, would be counted as collaborative publications between Iran, the US, and/or the UK.

This paper indicated that h-index of Iran’s publications has had a growing trend over ten 5-year periods. This means that along the substantial quantitative growth of publications, citation counts have increased to a certain degree, which confirms some previous reports on this [38,48,49]. The author and colleagues had previously reported on similar significant growing trends of 5-year h-indices of public health research publications in some other LMICs [2]. For instance, it was shown that the 5-year h-index of public health research publications of China had increased from 36 in the period 1996-2000 to 100 during 2006-2010. This figure for South Africa was 30 in the first 5-year period and was 32 for Brazil, while it increased to 78 publications in both countries for the period 2006-2010 [2].

Some may criticize the use of h-index for assessing citation counts [50]. First, one argument could be that since the maximum of h-index is the number of publications in a research unit, h-index is more strongly formed by publication counts rather than citation counts [51]. However, if the rise had been restricted to the quantity, it would have been impossible to see any improvements in the h-index over time. Second, another objection could be that h-index is insensitive to the total number of publications, thus it does not provide any information about the large proportion of publications that possibly have received minimal or zero citations [51]. This is a valid point and this paper did not aim to investigate improvements in the average citation counts, nor in citations per document. Nevertheless, what this paper could suggest is that in the long term, the increase in the quantity has promoted the number of publications that had a potential to receive a ‘reasonable’ number of citations. Third, many of the papers contributing to 5-year h-indices might be a result of international collaborations; even so, this is an achievement for a country that has been internationally isolated for four decades [46]. Finally, some may criticize the improved citation counts of the publications for having been self-citation in most cases. But the effect of author self-citations on global or national analyses is so insignificant that there is no need to exclude them [52].

This paper analysed the 155 h-core publications, among which ~ 70% were the product of an international collaboration. In general, papers with multi-national contribution often receive more citations [52]. Thirty-four of the identified most-cited papers had at least one Iranian co-author who was affiliated with international institutions. Iran has a high rate of brain drain, which is difficult to control given the country’s increasing unemployment rate among the educated population and the social and economic instability [47]. This study highlighted that one way to use the capacity of the emigrated Iranian academics could be provision of further opportunities for collaboration with them.

Two of the highly-cited papers had an international author affiliated with an Iranian university: Pahlavi University, which was renamed to Shiraz University of Medical Sciences following the 1979 Islamic Revolution. This university was modelled after American schools, and used several international academics aiming to educate Iranian medical doctors [47]. The majority – if not all – of international academics left Iran following the 1979 Revolution.

Studying the most-cited papers suggests that the GBD studies had a significant share in Iran’s h-core papers. The majority of these papers had an internationally well-known Iranian collaborator affiliated with TUMS, and were published in *The Lancet*. In terms of the research area, GBD papers should be attributed to “public health” area, but WoS attributes the papers that are published in general medical journals – eg, *The Lancet* – to “General Internal Medicine”. Across the 155 h-core papers, five prolific Iranian authors who are based abroad were identified. It appears that these academics have interest and ability in establishing successful collaborations with their peers in Iran; thus, further collaboration with them should be encouraged. In terms of authors of the 48 “only Iranian” papers, five researchers had contributed to publishing at least three impactful papers (by relying on Iranian research resources). It should be ensured that these academics would receive adequate resources to continue their research activities.

Regarding where the 48 “only Iranian” papers were published, it should be highlighted that they were not published in journals with very high IFs (IF ranged between 0.4 and 8.3 with a median of 2.3). The use of journal-level metrics for evaluating individual publications and their authors is rejected by many, because the distribution of citations over the publications in a journal is highly skewed [53]. However, still many decisions by funding bodies or academic employers are made upon IF [52]. This study showed that at least in the case of clinical, biomedical, and public health research publications that had only relied on Iranian resources, the likelihood of getting cited in the future was independent of the IF of the journals where they were published. Another finding worth noticing is that while the proportion of review articles among the total publications of Iran was small (2.7%), 37.5% of the ‘only Iranian’ highly-cited papers were review articles. In general, review articles tend to attract more citations than other document types [52].

The 48 “only Iranian” h-core papers were categorized into basic, clinical, and public health research and the majority were in basic sciences. This should trigger further thinking in Iranian medical research policy-makers regarding how best the financial and human resources in clinical, biomedical, and public health research should be distributed. For instance, one approach could be to always allocate a certain proportion of resources to basic sciences, where some potential for attracting recognition seems to exist. Particularly, supporting publication of review articles in basic sciences could be a strategy when resources are scarce. Then, in deciding where to invest the rest, one approach could be identifying the neglected research areas across the total publications and investigate whether research in some areas is less promoted or less supported. Some of the advantages and limitations of this study are presented in **Box 4**.

Box 4 **Advantages and limitations of this study**

This study had several advantages as follows: (i) careful inclusion of research areas to increase the coverage, while maintaining the specificity; (ii) using the ‘country filed tag’ rather than the ‘address’ filter, which is more specific; (iii) using ‘organization-enhanced’ for analysing the institutions instead of ‘organization’, which the former searches the unified name variant of affiliations; (iv) using a novel approach to find different types of collaborations across the Iranian h-core papers; and (v) studying the publications over half a century.

This study had limitations too. One limitation originates in the way WoS attributes research areas to each document, which is done automatically based on scope of the journals where the documents are published. One consequent problem is that some documents may be included because of having been published in journals with a clinical, biomedical, and/or public health scope while the articles’ content may be irrelevant. For the same reason, the retrieved number of publications in each research area could be misleading. It should also be noted that this study only included publications from WoS CC, which are not representative of all the publications arising from Iran. Finally, although investigation of the country of origin of the authors was undertaken very carefully (based on the language of the names and/or searching the study and work background of the authors on the Web) and in understanding different types of collaborations (eg, deciding on whether a project was a consortium) across the h-core papers, this approach could be susceptible to subjective assessment.

The study presented in this paper highlighted the strong research capacity that in Iran lies in clinical, biomedical, and public health fields; ~ 30% of all the publications were in these areas. Fairly similar results had been indicated in analyses using Scopus database [48]. This paper also showed that some of these publications, although relying only on Iranian resources, were internationally widely cited and recognized. Such capacity can be strengthened and better used with further investment [31]. Currently, Iran’s investment in health research is limited. Iran’s GERD has rarely surpassed 0.5% [31]; it was 0.3% in 2014, of which only a small fraction was allocated to health [31].

Considering such limited investments, Iran’s endeavours towards improving the quantity, the impact and the quality of health research publications is admirable [35,54]. What requires further attention is to first, be aware of the challenges posed by using bibliometric tools in assessment of research activities. This paper pointed out the misleading allocation of research areas of documents in WoS; and highlighted that journal IFs cannot be representative of the citation impact of individual papers. Second, the limitations of bibliometric tools in assessment of research output and/or impact should be considered [55,56]. One inherent limitation of relying only on bibliometrics is that scientific outputs other than papers – eg, clinical guidelines, policy documents, or data sets - could be neglected [53,55,56]. Similarly, it is likely that studies with less chances of publication in prestigious journals – despite having great potentials of leading to societal, economic, or health impacts – would be undervalued, both by the funders and the researchers.

While increased health research investment is recommended to better use the existing health research capacity in Iran, the investments should be governed in a way that resources would be used efficiently. It is of much interest to study how much of Iran’s vast amount of research publications in clinical-/bio-medicine and public health had been aligned with health needs of the society. Furthermore, considering that ‘producing research outputs’ is only part of one of the functions of a health research system, it is important to understand how other functions of Iran’s health research system have changed over this 50-year period. Finally, drawing on the findings of this study, the following suggestions could be shared with other LMICs who are to improve their health research output: (i) invest in developing national journals and in supporting them to get indexed in international citation databases for increased international visibility of publications [21,48]; this could help with early identification of potential existing problems in research practices, thus would help with addressing the problems in time; (ii) provide opportunities for collaboration with academic nationals who have emigrated to developed countries, particularly support collaborations with those who have already proved competency and interest in establishing successful collaborations with their peers back home; (iii) identify and support researchers who are able to produce high-quality research output while relying only on resources from LMICs; (iv) in the times when resources for conducting high-quality original basic research are limited promote publication of high-quality review articles; and finally, (v) avoid over-relying on bibliometrics in academic assessment practices, particularly be aware of the challenges of using journal IF for evaluation of individual papers, and/or researchers.

## CONCLUSIONS

The number of clinical, biomedical, and public health research publications in Iran has significantly increased over the last 5 decades. The output in certain fields, such as pharmacology research has been greater. It seems that, in the long term, the quantitative increase has promoted the number of publications that had a potential to receive a relatively high number of citations. The majority of the highly-cited papers from Iran have been the product of international collaborations, many of the collaborations had become possible with the contribution of Iranian academics abroad. Regarding the Iranian papers that had only relied on national resources, the likelihood of getting cited in the future had been independent of the IF of the journal where the papers were published. The Iranian science policy-makers are encouraged to (i) support the researchers and institutions that have proved research capacity; (ii) direct further resources towards research areas and/or institutions that are lagging behind; (iii) facilitate further international collaboration with the academics and/or institutions that have shown the capacity for conducting successful research projects with Iran.
