# Supplementary material for: 50 years of Iranian clinical, biomedical, and public health research: a bibliometric analysis of the Web of Science Core Collection (1965-2014)
Source: J Glob Health. 2018 Jul 30;8(2):020701. doi: 10.7189/jogh.08.020701 (PMC6076565; doi:10.7189/jogh.08.020701)
Supplement: Online Supplementary Document [file jogh-08-020701-s001.zip › Mansoori_OSD_1.docx]

**Supplementary Tables**

**50 Years of Iranian clinical, biomedical, and public health research: A bibliometric analysis of the Web of Science Core Collection (1965-2014)**

Parisa Mansoori PharmD^1^

*^1^Centre for Global Health Research, Usher Institute of Population Health Sciences and Informatics, University of Edinburgh, Edinburgh, UK EH8 9AG*

**Table S1:** List of the 48 included research areas

‘Research Areas’ is a classification scheme aiming to be the indexing backbone by which all WoS products have their content classified. The forty-eight included research areas were the following:

PEDIATRICS OR TROPICAL MEDICINE OR UROLOGY NEPHROLOGY OR ENDOCRINOLOGY METABOLISM OR PATHOLOGY OR REPRODUCTIVE BIOLOGY OR RADIOLOGY NUCLEAR MEDICINE MEDICAL IMAGING OR TOXICOLOGY OR PHARMACOLOGY PHARMACY OR LIFE SCIENCES BIOMEDICINE OTHER TOPICS OR ALLERGY OR RESEARCH EXPERIMENTAL MEDICINE OR OBSTETRICS GYNECOLOGY OR PHYSIOLOGY OR GENERAL INTERNAL MEDICINE OR MICROBIOLOGY OR BIOCHEMISTRY MOLECULAR BIOLOGY OR PSYCHIATRY OR HEMATOLOGY OR NEUROSCIENCES NEUROLOGY OR MEDICAL LABORATORY TECHNOLOGY OR GASTROENTEROLOGY HEPATOLOGY OR HEALTH CARE SCIENCES SERVICES OR ORTHOPEDICS OR CELL BIOLOGY OR VIROLOGY OR PUBLIC ENVIRONMENTAL OCCUPATIONAL HEALTH OR GENETICS HEREDITY OR RHEUMATOLOGY OR BIOTECHNOLOGY APPLIED MICROBIOLOGY OR RESPIRATORY SYSTEM OR INFECTIOUS DISEASES OR IMMUNOLOGY OR TRANSPLANTATION OR NUTRITION DIETETICS OR MYCOLOGY OR VETERINARY SCIENCES OR SURGERY OR DENTISTRY ORAL SURGERY MEDICINE OR OPHTHALMOLOGY OR OTORHINOLARYNGOLOGY OR DERMATOLOGY OR REHABILITATION OR NURSING OR INTEGRATIVE COMPLEMENTARY MEDICINE OR ONCOLOGY OR PARASITOLOGY OR CARDIOVASCULAR SYSTEM CARDIOLOGY

| **Table S2:** List of the 155 Iranian h-core publications (1965-2014), indexed in Web of Science Core Collection, ranked by their year of publication, and then by citation count within each year  *Citations were calculated until 28 February, 2018. Citation counts depend on the year of publication and the research field. Higher or lower number of citations to the articles of this list does not represent any superiority/inferiority. | | | | |
| --- | --- | --- | --- | --- |
| **No.** | **Authors** | **Title** | **Biobliographical Information** | **Citation Count*** |
| 1 | Reinhold JG, Nasr K, Lahimgarzadeh A, et al. | Effects of purified phytate and phytate-rich bread upon metabolism of zinc, calcium, phosphorus, and nitrogen in man | The Lancet **1973**;1(7798):283-288 | 237 |
| 2 | Reinhold JG, Ismailbeigi F, Faradji B | Fiber vs phytate as determinant of availability of calcium, zinc and iron of breadstuffs | Nutrition Reports International **1975**;12(2):75-85 | 179 |
| 3 | Reinhold JG, Faradji B, Abadi P, et al. | Decreased absorption of calcium, magnesium, zinc and phosphorus by humans due to increased fiber and phosphorus consumption as wheat bread | Journal of Nutrition **1976**;106(4):493-503 | 314 |
| 4 | Cook-Mozaffari PJ, Azordegan F, Day NE, et al | Oesophageal cancer studies in the Caspian Littoral of Iran: results of a case-control study | British Journal of Cancer **1979**;39(3):293-309 | 201 |
| 5 | Lee SJ, Monsef M, Torabinejad M. | Sealing ability of a mineral trioxide aggregate for repair of lateral root perforations | Journal of Endodontics **1993**;19(11):541-544 | 363 |
| 6 | Torabinejad M , Hong CU, Lee SJ, et al. | Investigation of mineral trioxide aggregate for root-end filling in dogs | Journal of Endodontics **1995**;21(12):603-608 | 235 |
| 7 | Torabinejad M, Rastegar AF, Kettering JD, et al. | Bacterial leakage of mineral trioxide aggregate as a root-end filling material | Journal of Endodontics **1995**;21(3):109-112 | 207 |
| 8 | Wilson RC, Krozowski ZS, Li K, et al. | A mutation in the HSD11B2 gene in a family with apparent mineralocorticoid excess | Journal of Clinical Endocrinology & Metabolism **1995**;80(7):2263-2266 | 189 |
| 9 | Singh RB, Niaz MA, Sharma JP, et al. | Randomized, double-blind, placebo-controlled trial of fish oil and mustard oil in patients with suspectedacute myocardial infarction: the Indian experiment of infarct survival--4 | Cardiovascular drugs and Therapy **1997**;11(3):485-491 | 342 |
| 10 | Berden JHM, Madias NE, Broumand B, et al. | Lupus nephritis – Discussion | Kidney International **1997**;52(2):538-558 | 159 |
| 11 | Sharifi I, FeKri AR, Aflatonian MR, et al. | Randomised vaccine trial of single dose of killed Leishmania major plus BCG against anthroponoticcutaneous leishmaniasis in Bam, Iran | The Lancet **1998**;351(9115):1540-1543 | 165 |
| 12 | Hurvitz JR, Suwairi WM, Van Hul W, et al. | Mutations in the CCN gene family member WISP3 cause progressive pseudorheumatoid dysplasia | Nature Genetics **1999**;23(1):94-98 | 163 |
| 13 | Sadeghi H, Allard P, Prince F, et al. | Symmetry and limb dominance in able-bodied gait: a review | Gait & Posture **2000**;12(1):34-45 | 391 |
| 14 | Salehizadeh H, Shojaosadati SA | Extracellular biopolymeric flocculants. Recent trends and biotechnological importance | Biotechnology Advances **2001**; 19(5):371-385 | 264 |
| 15 | Montazeri A, Milroy R, Hole D, et al. | Quality of life in lung cancer patients - As an important prognostic factor | Lung Cancer **2001**;31(2-3):233-240 | 159 |
| 16 | Galati G, Sabzevari O, Wilson JX, et al. | Prooxidant activity and cellular effects of the phenoxyl radicals of dietary flavonoids and other polyphenolics | Toxicology **2002;**177(1):91-104 | 344 |
| 17 | Azizi F, Rahmani M, Emami H, et al. | Cardiovascular risk factors in an Iranian urban population: Tehran Lipid and Glucose Study (Phase 1) | Sozial- und Präventivmedizin **2002**;47(6):408-426 | 269 |
| 18 | Ghassemi H, Harrison G, Mohammad K | An accelerated nutrition transition in Iran | Public Health Nutrition **2002**;5(1A):149-155 | 233 |
| 19 | Ghiassi-nejad M, Mortazavi SM, Cameron JR, et al. | Very high background radiation areas of Ramsar, Iran: Preliminary biological studies | Health Physics **2002**;82(1):87-93 | 168 |
| 20 | Javanmardi J, Stushnoff C, Locke E, et al. | Antioxidant activity and total phenolic content of Iranian Ocimum accessions | Food Chemistry **2003**;83(4):547-550 | 306 |
| 21 | Azizi, F, Salehi, P, Etemadi, A, et al. | Prevalence of metabolic syndrome in an urban population: Tehran Lipid and Glucose Study | Diabetes Research and Clinical Practice **2003**;61(1):29-37 | 275 |
| 22 | Chen L, Lee L, Kudlow BA, et al. | LMNA mutations in atypical Werner's syndrome | The Lancet **2003**;362(9382):440-445 | 251 |
| 23 | Abdollahi M, Cushman M, Rosendaal FR | Obesity: risk of venous thrombosis and the interaction with coagulation factor levels and oral contraceptive use | Thrombosis and Haemostasis **2003**;89(3):493-498 | 250 |
| 24 | A Vessal M, Hemmati M, Vasei M | Antidiabetic effects of quercetin in streptozocin-induced diabetic rats | Comparative Biochemistry and Physiology - Part C: Toxicology & Pharmacology **2003**;135(3):357-364 | 238 |
| 25 | Torabinejad M, Khademi AA, Babagoli J, et al. | A new solution for the removal of the smear layer | Journal of Endodontics **2003**; 29(3):170-175 | 227 |
| 26 | Khateri S, Ghanei M, Keshavarz S, et al. | Incidence of lung, eye, and skin lesions as late complications in 34,000 Iranians with wartime exposure to mustard agent | Journal of Occupational and Environmental Medicine **2003**;45(11):1136-1143 | 184 |
| 27 | Akhgari M, Abdollahi M, Kebryaeezadeh A, et al. | Biochemical evidence for free radical-induced lipid peroxidation as a mechanism for subchronic toxicity of malathion in blood and liver of rats | Human & Experimental Toxicology **2003**;22(4): 205-211 | 182 |
| 28 | Zendehdel K, Nyren O, Ostenson CG, et al. | Cancer incidence in patients with type 1 diabetes mellitus: A population-based cohort study in Sweden | Journal of the National Cancer Institute **2003**;95(23):1797-1800 | 169 |
| 29 | Sarraf-Zadegan N, Sadri G, Afzali HM, et al. | Isfahan Healthy Heart Programme: a comprehensive integrated community-based programme for cardiovascular disease prevention and control. Design, methods and initial experience | Acta cardiologica **2003**;95(4):309-320 | 157 |
| 30 | Muzha I, Filipi N, Lede R, et al. | Effect of intravenous corticosteroids on death within 14 days in 10008 adults with clinically significant head injury (MRC CRASH trial): randomised placebo-controlled trial | The Lancet **2004**;364(9442):1321-1328 | 470 |
| 31 | Abdollahi M, Ranjbar A, Shadnia S, et al. | Pesticides and oxidative stress: a review | Medical Science Monitor **2004**;10(6): RA141-RA147 | 312 |
| 32 | Almasirad A, Tabatabai SA, Faizi M, et al. | Synthesis and anticonvulsant activity of new 2-substituted-5-[2-(2-fluorophenoxy)phenyl]-1,3,4-oxadiazoles and 1,2,4-triazoles | Bioorganic & Medicinal Chemistry Letters **2004**;14(4):6057-6059 | 224 |
| 33 | Siebenrock KA, Wahab KHA, Werlen S, et al. | Abnormal extension of the femoral head epiphysis as a cause of cam impingement | Clinical Orthopaedics and Related Research **2004**;418:54-60 | 209 |
| 34 | Ahmadian G, Ju W, Liu LD, et al. | Tyrosine phosphorylation of GluR2 is required for insulin-stimulated AMPA receptor endocytosis and LTD | EMBO Journal **2004**;23(5):1040-1050 | 165 |
| 35 | Noorbala AA, Yazdi SAB, Yasamy MT, et al. | Mental health survey of the adult population in Iran | British Journal of Psychiatry **2004**; 184: 70-73 | 165 |
| 36 | Reilly JJ, Armstrong J, Dorosty AR, et al. | Early life risk factors for obesity in childhood: cohort study | British Medical Journal **2005**;330(7504):1357-1359 | 847 |
| 37 | Rahimi R, Nikfar S, Larijani B, et al. | A review on the role of antioxidants in the management of diabetes and its complications | Biomedicine & Pharmacotherapy **2005**;59(7):365-373 | 411 |
| 38 | Enayati AA, Ranson H, Hemingway J | Insect glutathione transferases and insecticide resistance | Insect Molecular Biology **2005**; 14(1):3-8 | 353 |
| 39 | Montazeri A, Goshtasebi A, Vahdaninia M, et al. | The Short Form Health Survey (SF-36): translation and validation study of the Iranian version | Quality of Life Research **2005**;14(3):875-882 | 319 |
| 40 | Abdolmaleky, HM; Cheng, KH; Russo, A; et al. | Hypermethylation of the reelin (RELN) promoter in the brain of schizophrenic patients: A preliminary report | American Journal of Medical Genetics Part B: Neuropsychiatric Genetics **2005**;134B(1):60-66 | 267 |
| 41 | Azadbakht L, Mirmiran P, Esmailzadeh A, et al. | Beneficial effects of a Dietary Approaches to Stop Hypertension eating plan on features of the metabolic syndrome | Diabetes Care **2005**;28(12):2823-2831 | 251 |
| 42 | Azadbakht L, Mirmiran P, Esmailzadeh A, et al. | Dairy consumption is inversely associated with the prevalence of the metabolic syndrome inTehranian adults | American Journal of Clinical Nutrition **2005**;82(3):523-530 | 222 |
| 43 | Enver T, Soneji S, Joshi C, et al. | Cellular differentiation hierarchies in normal and culture-adapted human embryonic stem cells | Human Molecular Genetics **2005**;14(21):3129-3140 | 195 |
| 44 | Bertolote JM, Fleischmann A, De Leo D | Suicide attempts, plans, and ideation in culturally diverse sites: the WHOSUPRE-MISS community survey | Psychological Medicine **2005**;35(10):1457-1465 | 176 |
| 45 | Bonab MM, Alimoghaddam K, Talebian F, et al. | Aging of mesenchymal stem cell in vitro | BMC Cell Biology **2006**;7(14) | 360 |
| 46 | Safavi A, Maleki N, Moradlou O, et al. | Simultaneous determination of dopamine, ascorbic acid, and uric acid using carbon ionic liquid electrode | Analytical Biochemistry **2006**;359(2):224-229 | 301 |
| 47 | Barter, PJ; Ballantyne, CM; Carmena, R; et al. | Apo B versus cholesterol in estimating cardiovascular risk and in guiding therapy: report of the thirty-person/ten-country panel | Journal of Internal Medicine **2006**;259(3):247-258 | 280 |
| 48 | Abdolmaleky HM, Cheng KH, Faraone SV, et al. | Hypomethylation of MB-COMT promoter is a major risk factor for schizophrenia and bipolar disorder | Human Molecular Genetics **2006**;15(21):3132-3145 | 276 |
| 49 | Pourmorad F, Hosseinimehr SJ, Shahabimajd N | Antioxidant activity, phenol and flavonoid contents of some selected Iranian medicinal plants | African Journal of Biotechnology **2006**;5(11):1142-1145 | 266 |
| 50 | Esmaillzadeh A, Kimiagar M, Mehrabi Y, et al. | Fruit and vegetable intakes, C-reactive protein, and the metabolic syndromes | The American Journal of Clinical Nutrition **2006**;84(6):1489-1497 | 235 |
| 51 | Went P, Vasei M, Bubendorf L, et al. | Frequent high-level expression of the immunotherapeutic target Ep-CAM in colon, stomach, prostate and lung cancers | British Journal of Cancer **2006**;94(1):128-135 | 216 |
| 52 | Eidi A, Eidi M, Esmaeili E | Antidiabetic effect of garlic (Allium sativum L.) in normal and streptozotocin-induced diabetic rats | Phytomedicine **2006**;13(9-10):624-629 | 163 |
| 53 | Hamidi M, Azadi A, Rafiei P | Pharmacokinetic consequences of pegylation | Drug Delivery **2006**;13(6):399-409 | 161 |
| 54 | Shahverdi AR, Fakhimi A, Shahverdi HR, et al. | Synthesis and effect of oxidative a new  dinar nanoparticles on the antibacterial activity of different antibiotics against Staphylococcus aureus and Escherichia coli | Nanomedicine: Nanotechnology, Biology and Medicine **2007**;3(2):168-171 | 531 |
| 55 | Janghorbani M, Van Dam RM, Willett WC, et al. | Systematic review of type 1 and type 2 diabetes mellitus and risk of fracture | American Journal of Epidemiology **2007**;166(5):495-505 | 459 |
| 56 | Hedayati MT, Pasqualotto AC, Warn PA, et al. | Aspergillus flavus: human pathogen, allergen and mycotoxin producer | Microbiology **2007**;153(Pt6):1677-1692 | 319 |
| 57 | Goodman Robert L, Lehman, Michael N, Smith Jeremy T, et al. | Kisspeptin neurons in the arcuate nucleus of the ewe express both dynorphin a and neurokinin B | Endocrinology **2007**;148(12):5752-5760 | 319 |
| 58 | Kelishadi R | Childhood overweight, obesity, and the metabolic syndrome in developing countries | Epidemiologic Reviews **2007**;29:62-76 | 308 |
| 59 | Farooqi IS, Wangensteen T, Collins S, et al. | Clinical and molecular genetic spectrum of congenitaldeficiency of the leptin receptor | New England Journal of Medicine **2007**;365(3):237-247 | 303 |
| 60 | Rezaie A, Parker RD, Abdollahi M | Oxidative stress and pathogenesis of inflammatory bowel disease: An epiphenomenon or the cause? | Digestive Diseases and Sciences **2007**;52(9):2015-2021 | 284 |
| 61 | Shahverdi AR, Minaeian S, Shahverdi HR, et al. | Rapid synthesis of silver nanoparticles using culture supernatants of *Enterobacteria*: A novel biological approach | Process Biochemistry **2007**;42(5):919-923 | 279 |
| 62 | Klein C, Grudzien M, Appaswamy G, et al. | HAX1 deficiency causes autosomal recessive severe congenital neutropenia (Kostmann disease) | Nature Genetics **2007**;39(1):86-92 | 264 |
| 63 | Akhoondi S, Sun D, von der Lehr N, et al. | FBXW7/hCDC4 is a general tumor suppressor in human cancer | Cancer Research **2007**;67(19):9006-9012 | 240 |
| 64 | Moradali MF, Mostafavi, H, Ghods S, et al | Immunomodulating and anticancer agents in the realm of macromycetes fungi (macrofungi) | International Immunopharmacology **2007**;7(6):701-724 | 240 |
| 65 | Hosseinimehr SJ | Foundation review: Trends in the development of radioprotective agents | Drug Discovery Today **2007**;12(19-20):794-805 | 225 |
| 66 | Rhead JL, Letley DP, Mohammadi M, et al. | A new Helicobacter pylori vacuolating cytotoxin determinant, the intermediate region, is associated with gastric cancer | Gastroenterology **2007**;133(3):926-936 | 215 |
| 67 | Kiani R, Esteky H, Mirpour K, et al. | Object category structure in response patterns of neuronal population in monkey inferior temporal cortex | Journal of Neurophysiology **2007**;97(6):4296-4309 | 214 |
| 68 | Esmaillzadeh A, Kimiagar M, Mehrabi Y, et al. | Dietary patterns, insulin resistance, and prevalence of the metabolic syndrome in women | The American Journal of Clinical Nutrition **2007**;85(3):910-918 | 207 |
| 69 | Atlasi Y, Mowla SJ, Ziaee SA, Bahrami AR | OCT-4, an embryonic stem cell marker, is highly expressed in bladder cancer | International Journal of Cancer **2007**;120(7):1598-1602 | 173 |
| 70 | Akhondzadeh S, Tabatabaee M, Amini H, et al | Celecoxib as adjunctive therapy in schizophrenia: A double-blind, randomized and placebo-controlled trial | Schizophrenia Research **2007**;90(1-3):179-185 | 169 |
| 71 | Esmaillzadeh A, Kimiagar M, Mehrabi Y, et al. | Dietary patterns and markers of systemic inflammation among Iranian women | The Journal of Nutrition **2007**;137(4):992-998 | 169 |
| 72 | Mohamadnejad M, Alimoghaddam, K, Mohyeddin-Bonab M, et al. | Phase 1 trial of autologous bone marrow mesenchymal stem cell transplantation in patients with decompensated liver cirrhosis | Archives of Iranian Medicine **2007**;10(4):459-466 | 165 |
| 73 | Holmes C, Boche D, Wilkinson D, et al. | Long-term effects of A beta(42) immunisation in Alzheimer's disease: follow-up of a randomised, placebo-controlled phase I trial | The Lancet **2008**;372(9634):216-223 | 860 |
| 74 | Hamidi M, Azadi A, Rafiei P | Hydrogel nanoparticles in drug delivery | Advanced Drug Delivery Reviews **2008**;60(15):1638-1649 | 778 |
| 75 | Asl MN, Hosseinzadeh H | Review of pharmacological effects of Glycyrrhiza sp and its bioactive compounds | Phytotherapy Research **2008**;22(6):709-724 | 433 |
| 76 | Kriegeskorte N, Mur M, Ruff DA, et al. | Matching Categorical Object Representations in Inferior Temporal Cortex of Man and Monkey | Neuron **2008**;60(6):1126-1141 | 419 |
| 77 | McQueen MJ, Hawken S, Wang X, et al. | Lipids, lipoproteins, and apolipoproteins as risk markers of myocardial infarction in 52 countries (the INTERHEART study): a case-control study | The Lancet **2008**;372(9634):224-233 | 393 |
| 78 | Montazeri A | Health-related quality of life in breast cancer patients: A bibliographic review of the literature from 1974 to 2007 | Journal of Experimental & Clinical Cancer Research **2008**;27:32 | 274 |
| 79 | Jouyban A | Review of the cosolvency models for predicting solubility of drugs in water-cosolvent mixtures | Journal of Pharmacy and Pharmaceutical Sciences **2008**;11(1):32-57 | 239 |
| 80 | Imanshahidi M, Hosseinzadeh H | Pharmacological and therapeutic effects of Berberis vulgaris and its active constituent, berberine | Phytotherapy Research **2008**;22(8):999-1012 | 224 |
| 81 | Azarmi S, Roa WH, Löbenberg R | Targeted delivery of nanoparticles for the treatment of lung diseases | Advanced Drug Delivery Reviews **2008**;60(8):863-875 | 222 |
| 82 | Smith JT, Coolen LM, Kriegsfeld LJ, et al. | Variation in Kisspeptin and RFamide-Related Peptide ( RFRP) Expression and Terminal Connections to Gonadotropin-Releasing Hormone Neurons in the Brain: A Novel Medium for Seasonal Breeding in the Sheep | Endocrinology **2008**;149(11):5770-5782 | 218 |
| 83 | Niknejad H, Peirovi H, Jorjani, M, et al. | Properties of the amniotic membrane for potential use in tissue engineering | European Cells & Materials **2008**;15:88-99 | 217 |
| 84 | Gill P, Ghaemi A | Nucleic acid isothermal amplification technologies - A review | Nucleosides, Nucleotides and Nucleic Acids **2008**; 27(3):224-243 | 195 |
| 85 | Atlasi Y, Mowla SJ, Ziaee SA, et al. | OCT4 Spliced Variants Are Differentially Expressed in Human Pluripotent and Nonpluripotent Cells | Stem Cells **2008**;26(12):3068-3074 | 185 |
| 86 | Islami F, Kamangar F | Helicobacter pylori and Esophageal Cancer Risk: A Meta-analysis | Cancer Prevention Research **2008**;1(5):329-338 | 175 |
| 87 | Kim DH, Sabour S, Sagar, U N, et al. | Prevalence of Hypovitaminosis D in Cardiovascular Diseases (from the National Health and Nutrition Examination Survey 2001 to 2004) | American journal of cardiology **2008**;102(11):1540-1544 | 175 |
| 88 | Ghavami S, Rashedi I, Dattilo BM, et al. | S100A8/A9 at low concentration promotes tumor cell growth via RAGE ligation and MAP kinase-dependent pathway | Journal of Leukocyte Biology 2008;83(6):1484-1492 | 168 |
| 89 | Beitollahi H, Mazloum-Ardakani M, Ganjipour B, et al. | Novel 2,2 '-[1,2-ethanediylbis(nitriloethylidyne)]-bis-hydroquinone double-wall carbon nanotube paste electrode for simultaneous determination of epinephrine, uric acid and folic acid | Biosensors & Bioelectronics **2008**;24(3):362-368 | 168 |
| 90 | Esteghamati A, Gouya MM, Abbasi M, et al. | Prevalence of diabetes and impaired fasting glucose in the adult population of Iran - National Survey of Risk Factors for Non-Communicable Diseases of Iran | Diabetes Care **2008**;31(1):96-98 | 157 |
| 91 | Glocker EO, Hennigs A, Nabavi M, et al. | A Homozygous CARD9 Mutation in a Family with Susceptibility to Fungal Infections | The New England Journal of Medicine **2009**; 361(18):1727-1735 | 363 |
| 92 | Forsetlund L, Bjørndal A, Rashidian A, et al. | Continuing education meetings and workshops: effects on professional practice and health care outcomes | The Cochrane Database of Systematic Reviews **2009**;2:CD003030 | 358 |
| 93 | Ghavami S, Hashemi M, Ande SR, et al. | Apoptosis and cancer: mutations within caspase genes | Journal of Medical Genetics **2009**;46(8):497-510 | 331 |
| 94 | Soleimani M, Naderi S | A protocol for isolation and culture of mesenchymal stem cells from mouse bone marrow | Nature Protocols **2009**;4(1):102-106 | 316 |
| 95 | Velayati AA, Masjedi MR, Farnia P, et al. | Emergence of New Forms of Totally Drug-Resistant Tuberculosis Bacilli Super Extensively Drug-Resistant Tuberculosis or Totally Drug-Resistant Strains in Iran | Chest **2009**;136(2):420-425 | 304 |
| 96 | Huxley RR, Ansary-Moghaddam A, Clifton P, et al. | The impact of dietary and lifestyle risk factors on risk of colorectal cancer: A quantitative overview of the epidemiological evidence | International Journal of Cancer **2009**;125(1):171-180 | 289 |
| 97 | Azizi F, Ghanbarian A, Momenan AA, et al. | Prevention of non-communicable disease in a population in nutrition transition: Tehran Lipid and Glucose Study phase II | Trials **2009**;10(1):5 | 277 |
| 98 | Engelhardt KR, McGhee S, Winkler S, et al. | Large deletions and point mutations involving the dedicator of cytokinesis 8 (DOCK8) in the autosomal-recessive form of hyper-IgE syndrome | The Journal of Allergy and Clinical Immunology **2009**;124(6): 1289-302.e4 | 229 |
| 99 | Mousavi SM, Gouya MM, Ramazani R, et al. | Cancer incidence and mortality in Iran | Annals of Oncology **2009**;20(3):556-563 | 223 |
| 100 | Safavi A, Maleki N, Farjami E | Fabrication of a glucose sensor based on a novel nanocomposite electrode | Biosensors & Bioelectronics **2009**;24(6):1655-1660 | 218 |
| 101 | Monot M, Honoré N, Garnier T, et al. | Comparative genomic and phylogeographic analysis of Mycobacterium leprae | Nature Genetics **2009**;41(12):1282-9 | 205 |
| 102 | Kharaziha P, Hellström PM, Noorinayer B, et al. | Improvement of liver function in liver cirrhosis patients after autologous mesenchymal stem cell injection: a phase I-II clinical trial | European Journal of Gastroenterology & Hepatology **2009**;21(10):1199-205 | 202 |
| 103 | Montazeri A | Quality of life data as prognostic indicators of survival in cancer patients: an overview of the literature from 1982 to 2008 | Health and Quality of Life Outcomes **2009**;7:102 | 183 |
| 104 | Delavari A, Forouzanfar, MH, Alikhani S, et al. | First Nationwide Study of the Prevalence of the Metabolic Syndrome and Optimal Cutoff Points of Waist Circumference in the Middle East The National Survey of Risk Factors for Noncommunicable Diseases of Iran | Diabetes Care **2009**;32(6):1029-1097 | 181 |
| 105 | Meshkani R, Adeli K | Hepatic insulin resistance, metabolic syndrome and cardiovascular disease | Clinical Biochemistry **2009**;42(13-14):1331-1346 | 180 |
| 106 | Akhondzadeh S, Jafari S, Raisi F, et al. | Clinical trial of adjunctive celecoxib treatment in patients with major depression: a double blind and placebo controlled trial | Depression and Anxiety **2009**;26(7):607-611 | 171 |
| 107 | Ranjbar B, Gill P | Circular Dichroism Techniques: Biomolecular and Nanostructural Analyses- A Review | Chemical Biology & Drug Design **2009**;74(2):101-120 | 166 |
| 108 | Ghasemi K, Ghasemi Y, Ebrahimzadeh MA | Antioxidant activity, phenol and flavonoid contents of 13 citrus species peels and tissues | Pakistan Journal of Pharmaceutical Sciences **2009**;22(3):277-281 | 161 |
| 109 | Parirokh M, Torabinejad M | Mineral Trioxide Aggregate: A Comprehensive Literature Review-Part III: Clinical Applications, Drawbacks, and Mechanism of Action | Journal of Endodontics **2010**;36(3):400-413 | 350 |
| 110 | Parirokh M, Torabinejad M | Mineral Trioxide Aggregate: A Comprehensive Literature Review-Part I: Chemical, Physical, and Antibacterial Properties | Journal of Endodontics **2010**;36(1):16-27 | 321 |
| 111 | Bousquet J, Mantzouranis E, Cruz AA, et al. | Uniform definition of asthma severity, control, and exacerbations: Document presented for the World Health Organization Consultation on Severe Asthma | The Journal of Allergy and Clinical Immunology **2010**;126(5):926-938 | 304 |
| 112 | Torabinejad M, Parirokh M | Mineral Trioxide Aggregate: A Comprehensive Literature Review-Part II: Leakage and Biocompatibility Investigations | Journal of Endodontics **2010**;36(2):190-202 | 269 |
| 113 | Esmaeili M, Mohabatkar H, Mohsenzadeh S | Using the concept of Chou's pseudo amino acid composition for risk type prediction of human papillomaviruses | Journal of Theoretical Biology **2010**;263(2):203-209 | 258 |
| 114 | Mohabatkar H | Prediction of Cyclin Proteins Using Chou's Pseudo Amino Acid Composition | Protein and Peptide Letters **2010**;17(10):1207-1214 | 215 |
| 115 | de Beaucoudrey L, Samarina A, Bustamante J, et al. | Revisiting human IL-12Rβ1 deficiency: a survey of 141 patients from 30 countries | Medicine **2010**;89(6):381-402 | 169 |
| 116 | Grady CL, Protzner AB, Kovacevic N, et al | A multivariate analysis of age-related differences in default mode and task-positive networksacross multiple cognitive domains. | Cerebral Cortex **2010**; 20(6):1432-1447 | 156 |
| 117 | Mahmoudi M, Sant S, Wang B, et al. | Superparamagnetic iron oxide nanoparticles (SPIONs): Development, surface modification and applications in chemotherapy | Advanced Drug Delivery Reviews **2011**;63(1-2):24-46 | 696 |
| 118 | Stagnaro-Green A, Abalovich M, Alexander E, et al. | Guidelines of the American Thyroid Association for the Diagnosis and Management of Thyroid Disease During Pregnancy and Postpartum | Thyroid: official journal of the American Thyroid Association **2011**;21(10):1081-1125 | 665 |
| 119 | Yusuf S, Islam S, Chow CK, et al. | Use of secondary prevention drugs for cardiovascular disease in the community in high-income, middle-income, and low-income countries (the PURE Study): a prospective epidemiological survey | The Lancet **2011**;378(9798):1231-1243 | 411 |
| 120 | International Stem Cell Initiative, Amps K, Andrews PW, et al. | Screening ethnically diverse human embryonic stem cells identifies a chromosome 20 minimal amplicon conferring growth advantage | Nature Biotechnology **2011**;29(12):1132-44 | 224 |
| 121 | Simchi A, Tamjid E, Pishbin F, et al. | Recent progress in inorganic and composite coatings with bactericidal capability for orthopaedic applications | Nanomedicine: Nanotechnology, Biology and Medicine **2011**;7(1):22-39 | 208 |
| 122 | Mohabatkar H, Beigi MM, Esmaeili A | Prediction of GABA(A) receptor proteins using the concept of Chou's pseudo-amino acid composition and support vector machine | Journal of Theoretical Biology **2011**;281(1):18-23 | 183 |
| 123 | Fedirko V, Tramacere I, Bagnardi V | Alcohol drinking and colorectal cancer risk: an overall and dose-response meta-analysis of published studies | Annals of Oncology **2011**;22(9):1958-1972 | 172 |
| 124 | Hawksworth DL, Crous PW, Redhead SA, et al. | The Amsterdam Declaration on Fungal Nomenclature | IMA Fungus **2011**;2(1):105-112 | 169 |
| 125 | Jadidi-Niaragh F, Mirshafiey A. | Th17 Cell, the New Player of Neuroinflammatory Process in Multiple Sclerosis | Scandinavian Journal of Immunology **2011**;74(1):1-13 | 164 |
| 126 | Dinarvand R, Sepehri N, Manoochehri S, et al. | Polylactide-co-glycolide nanoparticles for controlled delivery of anticancer agents | International Journal of Nanomedicine **2011**;6:877-895 | 156 |
| 127 | Lim SS, Vos T, Flaxman AD, et al. | A comparative risk assessment of burden of disease and injury attributable to 67 risk factors and risk factor clusters in 21 regions, 1990-2010: a systematic analysis for the Global Burden of Disease Study 2010 | The Lancet **2012**;380(9859):2224-2260 | 3,989 |
| 128 | Murray CJ, Vos T, Lozano R, et al. | Disability-adjusted life years (DALYs) for 291 diseases and injuries in 21 regions, 1990-2010: a systematic analysis for the Global Burden of Disease Study 2010 | The Lancet **2012**;380(9859):2197-2223 | 2,816 |
| 129 | Vos T, Flaxman AD, Naghavi M, et al. | Years lived with disability (YLDs) for 1160 sequelae of 289 diseases and injuries 1990-2010: a systematic analysis for the Global Burden of Disease Study 2010 | The Lancet **2012**;380(9859):2163-96 | 2,417 |
| 130 | Klionsky DJ, Abdalla FC, Abeliovich H, et al. | Guidelines for the use and interpretation of assays for monitoring autophagy | Autophagy **2012**;8(4):445-544 | 2,001 |
| 131 | Hajipour MJ, Fromm KM, Ashkarran, Ashkarran AA, et al. | Antibacterial properties of nanoparticles | Trends in Biotechnology **2012**;30(10):499-511 | 516 |
| 132 | Salomon JA, Vos T, Hogan DR, et al. | Common values in assessing health outcomes from disease and injury: disability weights measurement study for the Global Burden of Disease Study 2010 | The Lancet **2012**;380(9859):2129-43 | 496 |
| 133 | Stevens GA, Singh GM, Lu Y, et al | National, regional, and global trends in adult overweight and obesity prevalences | Population Health Metrics 2012;10(1):22 | 280 |
| 134 | Ahuja SD, Ashkin D, Avendano M, et al. | Multidrug Resistant Pulmonary Tuberculosis Treatment Regimens and Patient Outcomes: An Individual Patient Data Meta-analysis of 9,153 Patients | PloS Medicine **2012**;9(8):e1001300 | 255 |
| 135 | Bousquet J, Schünemann HJ, Samolinski B, et al. | Allergic Rhinitis and its Impact on Asthma (ARIA): Achievements in 10 years and future needs | The Journal of allergy and clinical immunology 2012;130(5):1049-1062 | 246 |
| 136 | Collaborative Group on Hormonal Factors in Breast Cancer | Menarche, menopause, and breast cancer risk: individual participant meta-analysis, including 118 964 women with breast cancer from 117 epidemiological studies | The Lancet Oncology **2012**;13(11):1141-1151 | 162 |
| 137 | Nabavi SM, Nabavi SF, Eslami S, et al. | In vivo protective effects of quercetin against sodium fluoride-induced oxidative stress in the hepatic tissue | Food Chemistry **2012**;132(2):931-935 | 156 |
| 138 | Davies C, Pan H, Godwin J, et al. | Long-term effects of continuing adjuvant tamoxifen to 10 years versus stopping at 5 years after diagnosis of oestrogen receptor-positive breast cancer: ATLAS, a randomised trial | The Lancet **2013**;381(9869):805-816 | 647 |
| 139 | Klattenhoff CA, Scheuermann JC, Surface LE, et al. | Braveheart, a Long Noncoding RNA Required for Cardiovascular Lineage Commitment | Cell **2013**;152(3):570-583 | 361 |
| 140 | Chow CK, Teo KK, Rangarajan S, et al | Prevalence, Awareness, Treatment, and Control of Hypertension in Rural and Urban Communities in High-, Middle-, and Low-Income Countries | JAMA **2013**;310(9):959-968 | 310 |
| 141 | Mostafalou S, Abdollahi M. | Pesticides and human chronic diseases: evidences, mechanisms, and perspectives | Toxicology and Applied Pharmacology **2013**;268(2):157-177 | 255 |
| 142 | Phillips AJ, Alves A, Abdollahzadeh J,et al | The Botryosphaeriaceae: genera and species known from culture | Studies in Mycology **2013**;76(1):51-167 | 206 |
| 143 | Karimi-Maleh H, Biparva P, Hatami M. | A novel modified carbon paste electrode based on NiO/CNTs nanocomposite and (9, 10-dihydro-9, 10-ethanoanthracene-11, 12-dicarboximido)-4-ethylbenzene-1, 2-diol as a mediator for simultaneous determination of cysteamine, nicotinamide adenine dinucleotide and folic acid | Biosensors and Bioelectronics **2013**;48:270-275 | 192 |
| 144 | Di Cesare M, Khang YH, Asaria P, et al. | Inequalities in non-communicable diseases and effective responses | The Lancet **2013**;381(9866):585-597 | 183 |
| 145 | Modabbernia A, Taslimi S, Brietzke E,et al | Cytokine alterations in bipolar disorder: a meta-analysis of 30 studies | Biological Psychiatry **2013**;74(1):15-25 | 171 |
| 146 | Ng M, Fleming T, Robinson M, et al. | Global, regional, and national prevalence of overweight and obesity in children and adults during 1980-2013: a systematic analysis for the Global Burden of Disease Study 2013 | The Lancet **2014**;384(9945):766-81 | 2,521 |
| 147 | Kassebaum NJ, Bertozzi-Villa A, Coggeshall MS, et al. | Global, regional, and national levels and causes of maternal mortality during 1990-2013: a systematic analysis for the Global Burden of Disease Study 2013 | The Lancet **2014**;384(9947):980-1004 | 410 |
| 148 | Murray CJ, Ortblad KF, Guinovart C1, et al | Global, regional, and national incidence and mortality for HIV, tuberculosis, and malaria during 1990-2013: a systematic analysis for the Global Burden of Disease Study 2013 | The Lancet **2014**;384(9947):1005-1070 | 330 |
| 149 | Wang H, Liddell CA, Coates MM, et al | Global, regional, and national levels of neonatal, infant, and under-5 mortality during 1990-2013: a systematic analysis for the Global Burden of Disease Study 2013 | The Lancet **2014**;384(9947):957-979 | 257 |
| 151 | O'Donnell M, Mente A, Rangarajan S, et al. | Urinary sodium and potassium excretion, mortality, and cardiovascular events | The New England Journal of Medicine **2014;** 371(7):612-623 | 249 |
| 150 | Ghavami S, Shojaei S, Yeganeh B, et al. | Autophagy and apoptosis dysfunction in neurodegenerative disorders | Progress in Neurobiology **2014**;112:24-49 | 238 |
| 152 | Muthuri SG, Venkatesan S, Myles PR, et al. | Effectiveness of neuraminidase inhibitors in reducing mortality in patients admitted to hospital with influenza A H1N1pdm09 virus infection: a meta-analysis of individual participant data | The Lancet Respiratory Medicine **2014**;2(5):395-404 | 205 |
| 153 | Mente A, O'Donnell MJ, Rangarajan S, et al. | Association of urinary sodium and potassium excretion with blood pressure | The New England Journal of Medicine **2014**; 371(7):601-611 | 196 |
| 154 | Yusuf S, Rangarajan S, Teo K, et al | Cardiovascular risk and events in 17 low-, middle-, and high-income countries | The New England Journal of Medicine **2014**; 371(9): 818-827 | 180 |
| 155 | Oryan A, Alidadi S, Moshiri A, et al. | Bone regenerative medicine: classic options, novel strategies, and future directions | Journal of Orthopaedic Surgery and Research **2014**:9(1):18 | 176 |

| **Table S3.** List of the 48 ‘only Iranian’ h-core publications (1965-2014), indexed in Web of Science Core Collection, ranked by their year of publication, and then by citation count within each year  *Citations were calculated until 28 February, 2018. Citation counts depend on the year of publication and the research field. Higher or lower number of citations to the articles of this list does not represent any superiority/inferiority. | | | | | | | |
| --- | --- | --- | --- | --- | --- | --- | --- |
| **No.** | **Authors** | **Title** | **Biobliographic Information** | **% of Citations Originating in Iran** | **Citation Count** | **Research Type** | **IF in the Papers’ Publication Year** |
| 1 | Salehizadeh H, Shojaosadati SA | Extracellular biopolymeric flocculants - Recent trends and biotechnological importance | Biotechnology Advances **2001**; 19(5):371-385 | 1.1% | 264 | Review - Basic | 1.568 |
| 2 | Azizi F, Rahmani M, Emami H, et al. | Cardiovascular risk factors in an Iranian urban population: Tehran Lipid and Glucose Study (Phase 1) | Sozial- und Präventivmedizin **2002**;47(6):408-426 | 95.6% | 269 | Public Health | 0.639 |
| 3 | Azizi F, Salehi P, Etemadi A, et al. | Prevalence of metabolic syndrome in an urban population: Tehran Lipid and Glucose Study | Diabetes Research and Clinical Practice **2003**;61(1):29-37 | 69.3% | 275 | Public health | 1.68 |
| 4 | A Vessal M, Hemmati M, Vasei M | Antidiabetic effects of quercetin in streptozocin-induced diabetic rats | Comparative Biochemistry and Physiology - Part C: Toxicology & Pharmacology **2003**;135(3):357-364 | 8.5% | 238 | Basic | 1.469 |
| 5 | Akhgari M, Abdollahi M, Kebryaeezadeh A, et al. | Biochemical evidence for free radical-induced lipid peroxidation as a mechanism for subchronic toxicity of malathion in blood and liver of rats | Human & Experimental Toxicology **2003**;22(4): 205-211 | 18.4% | 182 | Basic | 0.99 |
| 6 | Sarraf-Zadegan N, Sadri G, Afzali HM, et al. | Isfahan Healthy Heart Programme: a comprehensive integrated community-based programme for cardiovascular disease prevention and control. Design, methods and initial experience | Acta Cardiologica **2003**;95(4):309-320 | 89.4% | 157 | Public health | 0.38 |
| 7 | Abdollahi M, Ranjbar A, Shadnia S, et al. | Pesticides and oxidative stress: a review | Medical Science Monitor **2004**;10(6): RA141-RA147 | 21.5% | 312 | Review – Public Health | 1.595 |
| 8 | Almasirad A, Tabatabai SA, Faizi M, et al. | Synthesis and anticonvulsant activity of new 2-substituted-5-[2-(2-fluorophenoxy)phenyl]-1,3,4-oxadiazoles and 1,2,4-triazoles | Bioorganic & Medicinal Chemistry Letters **2004**;14(4):6057-6059 | 7.9% | 224 | Basic | 2.333 |
| 9 | Noorbala AA, Yazdi SAB, Yasamy MT, et al. | Mental health survey of the adult population in Iran | British Journal of Psychiatry **2004**; 184: 70-73 | 82.6% | 165 | Public health | 4.175 |
| 10 | Rahimi R, Nikfar S, Larijani B, et al. | A review on the role of antioxidants in the management of diabetes and its complications | Biomedicine & Pharmacotherapy **2005**;59(7):365-373 | 31.3% | 411 | Review – Clinical | 2.069 |
| 11 | Azadbakht L, Mirmiran P, Esmailzadeh A, et al. | Beneficial effects of a Dietary Approaches to Stop Hypertension eating plan on features of the metabolic syndrome | Diabetes Care **2005**;28(12):2823-2831 | 18.9% | 251 | Clinical | 7.844 |
| 12 | Azadbakht L, Mirmiran P, Esmailzadeh A, et al. | Dairy consumption is inversely associated with the prevalence of the metabolic syndrome in Tehranian adults | American Journal of Clinical Nutrition **2005**;82(3):523-530 | 26.4% | 222 | Public health | 5.853 |
| 13 | Safavi A, Maleki N, Moradlou O, et al. | Simultaneous determination of dopamine, ascorbic acid, and uric acid using carbon ionic liquid electrode | Analytical Biochemistry **2006**;359(2):224-229 | 33% | 301 | Basic | 2.948 |
| 14 | Bonab MM, Alimoghaddam K, Talebian F, et al. | Aging of mesenchymal stem cell in vitro | BMC Cell Biology **2006**;7(14) | 3.4% | 360 | Basic | 2.742 |
| 15 | Pourmorad F, Hosseinimehr SJ, Shahabimajd N | Antioxidant activity, phenol and flavonoid contents of some selected Iranian medicinal plants | African Journal of Biotechnology **2006**;5(11):1142-1145 | 13% | 266 | Basic | 0.45 |
| 16 | Eidi A, Eidi M, Esmaeili E | Antidiabetic effect of garlic (Allium sativum L.) in normal and streptozotocin-induced diabetic rats | Phytomedicine **2006**;13(9-10):624-629 | 13% | 163 | Basic | 1.403 |
| 17 | Hamidi M, Azadi A, Rafiei P | Pharmacokinetic consequences of pegylation | Drug Delivery **2006**;13(6):399-409 | 3.1% | 161 | Review - Basic | 1.424 |
| 18 | Shahverdi AR, Fakhimi A, Shahverdi HR, et al. | Synthesis and effect of silver nanoparticles on the antibacterial activity of different antibiotics against Staphylococcus aureus and Escherichia coli | Nanomedicine: Nanotechnology, Biology and Medicine **2007**;3(2):168-171 | 8.9% | 532 | Basic | 5.44 |
| 19 | Moradali MF, Mostafavi, H, Ghods S, et al | Immunomodulating and anticancer agents in the realm of macromycetes fungi (macrofungi) | International Immunopharmacology **2007**;7(6):701-724 | 0.8% | 240 | Review - Basic | 2.066 |
| 20 | Kelishadi R | Childhood overweight, obesity, and the metabolic syndrome in developing countries | Epidemiologic Reviews **2007**;29:62-76 | 41.3% | 308 | Review - Public health | 5.429 |
| 21 | Shahverdi AR, Minaeian S, Shahverdi HR, et al. | Rapid synthesis of silver nanoparticles using culture supernatants of *Enterobacteria*: A novel biological approach | Process Biochemistry **2007**;42(5):919-923 | 8.5% | 279 | Basic | 2.336 |
| 22 | Atlasi Y, Mowla SJ, Ziaee SA, Bahrami AR | OCT-4, an embryonic stem cell marker, is highly expressed in bladder cancer | International Journal of Cancer **2007**;120(7):1598-1602 | 11.6% | 173 | Basic | 4.555 |
| 23 | Hosseinimehr SJ | Foundation review: Trends in the development of radioprotective agents | Drug Discovery Today **2007**;12(19-20):794-805 | 13.9% | 226 | Review - Basic | 6.671 |
| 24 | Akhondzadeh S, Tabatabaee M, Amini H, et al | Celecoxib as adjunctive therapy in schizophrenia: A double-blind, randomized and placebo-controlled trial | Schizophrenia Research **2007**;90(1-3):179-185 | 14.2% | 169 | Clinical | 4.24 |
| 25 | Mohamadnejad M, Alimoghaddam, K, Mohyeddin-Bonab M, et al. | Phase 1 trial of autologous bone marrow mesenchymal stem cell transplantation in patients with decompensated liver cirrhosis | Archives of Iranian Medicine **2007**;10(4):459-466 | 12.6% | 165 | Clinical | JIF started since 2009 with 0.874 |
| 26 | Hamidi M, Azadi A, Rafiei P | Hydrogel nanoparticles in drug delivery | Advanced Drug Delivery Reviews **2008**;60(15):1638-1649 | 9.1% | 778 | Review – Basic | 8.287 |
| 27 | Asl MN, Hosseinzadeh H | Review of pharmacological effects of Glycyrrhiza sp and its bioactive compounds | Phytotherapy Research **2008**;22(6):709-724 | 5.4% | 433 | Review – Basic | 1.772 |
| 28 | Montazeri A | Health-related quality of life in breast cancer patients: A bibliographic review of the literature from 1974 to 2007 | Journal of Experimental and Clinical Cancer Research **2008**;27(1):32 | 3.9% | 274 | Review – Public health | 1.184 |
| 29 | Jouyban A | Review of the cosolvency models for predicting solubility of drugs in water-cosolvent mixtures | Journal of Pharmacy and Pharmaceutical Sciences **2008**;11(1):32-57 | 44.7% | 239 | Review – Basic | 1.887 |
| 30 | Imanshahidi M, Hosseinzadeh H | Pharmacological and therapeutic effects of Berberis vulgaris and its active constituent, berberine | Phytotherapy Research **2008**;22(8):999-1012 | 14.8% | 225 | Review – Basic | 1.772 |
| 31 | Gill P, Ghaemi A | Nucleic acid isothermal amplification technologies - A review | Nucleosides Nucleotides & Nucleic Acids **2008**; 27(3):224-243 | 3.6% | 195 | Review – Basic | 0.571 |
| 32 | Beitollahi H, Mazloum-Ardakani M, Ganjipour B, et al. | Novel 2,2 '-[1,2-ethanediylbis(nitriloethylidyne)]-bis-hydroquinone double-wall carbon nanotube paste electrode for simultaneous determination of epinephrine, uric acid and folic acid | Biosensors & Bioelectronics **2008**;24(3):362-368 | 65.6% | 168 | Basic | 5.149 |
| 33 | Soleimani M, Nadri S | A protocol for isolation and culture of mesenchymal stem cells from mouse bone marrow | Nature Protocols **2009**;4(1):102-106 | 6.4% | 317 | Basic | 6.335 |
| 34 | Mousavi SM, Gouya MM, Ramazani R, et al. | Cancer incidence and mortality in Iran | Annals of Oncology **2009**;20(3):556-563 | 93.7% | 223 | Public health | 5.647 |
| 35 | Safavi A, Maleki N, Farjami E | Fabrication of a glucose sensor based on a novel nanocomposite electrode | Biosensors & Bioelectronics **2009**;24(6):1655-1660 | 14.3% | 218 | Basic | 5.429 |
| 36 | Azizi F, Ghanbarian A, Momenan AA, et al and the Tehran Lipid and Glucose Study Group | Prevention of non-communicable disease in a population in nutrition transition: Tehran Lipid and Glucose Study phase II | Trials **2009**;10(1):5 | 97.7% | 277 | Public health | 2.02 |
| 37 | Delavari A, Forouzanfar, MH, Alikhani S, et al. | First Nationwide Study of the Prevalence of the Metabolic Syndrome and Optimal Cutoff Points of Waist Circumference in the Middle East The National Survey of Risk Factors for Noncommunicable Diseases of Iran | Diabetes Care **2009**;32(6):1029-1097 | 81.4% | 181 | Public health | 6.718 |
| 38 | Akhondzadeh S, Jafari S, Raisi F, et al. | Clinical trial of adjunctive celecoxib treatment in patients with major depression: a double blind and placebo controlled trial | Depression and Anxiety **2009**;26(7):607-611 | 12.9% | 171 | Clinical | 2.926 |
| 39 | Ranjbar B, Gill P | Circular Dichroism Techniques: Biomolecular and Nanostructural Analyses- A Review | Chemical Biology & Drug Design **2009**;74(2):101-120 | 16.3% | 166 | Review – Basic | 2.473 |
| 40 | Ghasemi K, Ghasemi Y, Ebrahimzadeh MA | Antioxidant activity, phenol and flavonoid contents of 13 citrus species peels and tissues | Pakistan Journal of Pharmaceutical Sciences **2009**;22(3):277-281 | 25.3% | 161 | Basic | 0.588 |
| 41 | Esmaeili M, Mohabatkar H, Mohsenzadeh S | Using the concept of Chou's pseudo amino acid composition for risk type prediction of human papillomaviruses | Journal of Theoretical Biology **2010**;263(2):203-209 | 6.6% | 258 | Basic | 2.371 |
| 42 | Mohabatkar H | Prediction of Cyclin Proteins Using Chou's Pseudo Amino Acid Composition | Protein and Peptide Letters **2010**;17(10):1207-1214 | 4.7% | 215 | Basic | 1.849 |
| 43 | Mohabatkar H, Beigi MM, Esmaeili A | Prediction of GABA(A) receptor proteins using the concept of Chou's pseudo-amino acid composition and support vector machine | Journal of Theoretical Biology **2011**;281(1):18-23 | 6.6% | 183 | Basic | 2.208 |
| 44 | Jadidi-Niaragh F, Mirshafiey A. | Th17 Cell, the New Player of Neuroinflammatory Process in Multiple Sclerosis | Scandinavian Journal of Immunology **2011**;74(1):1-13 | 16.6% | 164 | Review – Basic | 2.230 |
| 45 | Dinarvand R, Sepehri N, Manoochehri S, et al. | Polylactide-co-glycolide nanoparticles for controlled delivery of anticancer agents | International Journal of Nanomedicine **2011**;6:877-895 | 13.6% | 156 | Review – Basic | 3.13 |
| 46 | Nabavi SM, Nabavi SF, Eslami S, et al. | In vivo protective effects of quercetin against sodium fluoride-induced oxidative stress in the hepatic tissue | Food Chemistry **2012**;132(2):931-935 | 55% | 156 | Basic | 3.334 |
| 47 | Mostafalou S, Abdollahi M. | Pesticides and human chronic diseases: evidences, mechanism, and perspectives | Toxicology and Applied Pharmacology **2013**;268(2):157-177 | 15.8% | 255 | Review – Public health | 3.975 |
| 48 | Karimi-Maleh H, Biparva P, Hatami M. | A novel modified carbon paste electrode based on NiO/CNTs nanocomposite and (9, 10-dihydro-9, 10-ethanoanthracene-11, 12-dicarboximido)-4-ethylbenzene-1, 2-diol as a mediator for simultaneous determination of cysteamine, nicotinamide adenine dinucleotide and folic acid | Biosensors and Bioelectronics **2013**;48:270-275 | 63.5% | 192 | Basic | 6.541 |

| Table S4. Distribution of document types and research categories across the 48 ‘only Iranian’ h-core publications | | | |
| --- | --- | --- | --- |
|  | Original Article | Review Article | Total |
| Basic | 18 | 13 | 31 |
| Clinical | 4 | 1 | 5 |
| Public Health | 8 | 4 | 12 |
